# Digital Health Interventions to Reduce Cancer-Related Fatigue Among Adolescents and Young Adults: Scoping Review

**DOI:** 10.2196/68834

**Published:** 2025-10-21

**Authors:** Shanshan Jiang, Xiaoyu Yang, Xinying Yu

**Affiliations:** 1 Department of Pediatrics, Shengjing Hospital of China Medical University, 36 Sanhao Street, Heping District, Shenyang, 110004, China, 86 18940251860; 2 Department of Oncology, Shengjing Hospital of China Medical University, Shenyang, China

**Keywords:** digital health, adolescent, young adult, cancer-related fatigue, scoping review

## Abstract

**Background:**

Cancer-related fatigue is a common and significant symptom experienced by patients with cancer and survivors across all age groups, profoundly impacting their quality of life. Adolescents and young adults often encounter substantial academic, career, and personal demands, which pose unique challenges in managing this symptom and may have a more profound overall impact on their lives. While digital health interventions show considerable promise in managing cancer-related fatigue, few reviews have specifically addressed their use among adolescents and young adults.

**Objective:**

This scoping review aimed to identify and assess the types and effectiveness of digital health interventions in managing cancer-related fatigue among adolescents and young adults.

**Methods:**

A comprehensive literature search was conducted using the keywords “digital health,” “adolescent,” “young adult,” “fatigue,” and “neoplasms” across 6 databases: PubMed, CINAHL, PsycINFO, Embase, Cochrane Library, and Web of Science. The search included English-language publications from the inception of each database to August 2024. Two researchers independently screened the studies based on predetermined inclusion and exclusion criteria.

**Results:**

A total of 2965 articles were retrieved during the initial search, of which 10 (0.34%) satisfied the inclusion criteria of this review. The 10 included studies comprised 5 (50%) randomized controlled trials, 2 (20%) quasi-experimental studies, 2 (20%) mixed methods studies, and 1 (10%) cohort study. On the basis of the functions and forms of digital health interventions, the interventions included in this review were categorized into the following 5 types: dynamic health monitoring and feedback, automated online guidance and feedback, live remote coaching and instruction, gamified interventions, and robot-assisted interventions. Multiple studies (7/10, 70%) demonstrated that digital health interventions are effective in reducing cancer-related fatigue in adolescents and young adults and show potential in improving physical function and emotional well-being in this population.

**Conclusions:**

Digital health interventions overcome the time and spatial limitations of traditional treatments and provide holistic support across physical, psychological, and social domains. They hold significant potential to alleviate cancer-related fatigue in adolescents and young adults. Future research should integrate various fatigue measurement scales and conduct large-scale studies and long-term follow-ups to capture a more comprehensive range of fatigue experiences, validate these findings, and enhance the effectiveness of digital health interventions.

## Introduction

### Background

Cancer-related fatigue (CRF) is a widespread symptom that significantly diminishes quality of life. It is characterized by persistent physical, emotional, and cognitive fatigue that is not proportional to recent activities, profoundly impacting daily functioning [1]. To accurately evaluate this complex symptom, various scales have been developed in the literature, including unidimensional single-item scales, unidimensional multi-item scales, and multidimensional scales specifically designed for patients with cancer. Among these, the most widely used and extensively validated scales are the Functional Assessment of Cancer Therapy–Fatigue, the European Organisation for Research and Treatment of Cancer Quality of Life Questionnaire (fatigue subscale), and the Fatigue Questionnaire [2,3]. Epidemiological studies indicate that approximately 65% of patients with cancer are affected by CRF, with more than two-thirds of patients reporting that fatigue persists for at least 6 months and is moderate to severe. One-third of patients report that fatigue continues for several years after treatment [4]. Specifically, the prevalence of CRF is highest in patients undergoing cancer treatment before discharge and decreases within 6 months following the completion of acute treatment [5], and the incidence of fatigue is higher in patients with leukemia and lymphoma than in those with central nervous system tumors and solid tumors [6]. CRF is prevalent among patients with cancer and survivors across all age groups, but its severity differs among age groups [7]. Adolescents and young adults, defined as individuals aged 15 to 39 years, face the highest CRF severity [8]. They must cope with not only the physical and psychological demands of cancer treatment but also the pressures associated with major life stages, such as academic responsibilities, career initiation, social connections, and family dynamics [9-11]. These compounded pressures heighten both mental and physical stress, worsening CRF symptoms. The management of CRF primarily depends on nonpharmacological approaches, including modifications to activity levels, lifestyle improvements, and the promotion of mental health [12]. This process often requires comprehensive support, including treatment adherence, health behavior modification, self-management skills, physician-patient communication, and support from family and social networks. CRF requires long-term management. However, traditional CRF management is constrained by factors such as the availability of health care professionals, time, financial resources, environments, and facilities [13]. Furthermore, adolescents and young adults frequently lack sufficient understanding of their cancer treatment history and the risks associated with late effects. This, in combination with their busy schedules, often results in the neglect of CRF treatment [14]. Thus, there is an urgent need for effective and sustainable interventions for adolescents and young adults to assist them in effectively managing CRF.

As the application and popularity of digital health interventions (DHIs) continue to grow, numerous studies have demonstrated that DHIs have potential in supporting the symptom management of patients with cancer [15,16]. *Digital health technologies* refer to the application of digital technology to the health sector, with examples including telemedicine; robotics; and mobile health supported by smartphones, wearable devices, mobile apps, and various monitoring technologies [17]. Studies have shown that the application of these technologies combined with interventions in CRF management is of great importance. Telemedicine services allow patients to consult with physicians via video, phone, or other digital platforms, obtaining personalized guidance on fatigue management [18]. This reduces the time and financial burden associated with seeking medical care and enhances patient accessibility. Robotics technology can offer precise movement guidance and support throughout the patient’s rehabilitation, assisting patients in more effectively strengthening muscles and restoring mobility, thereby helping alleviate CRF symptoms [19]. Mobile health technologies, using various mobile devices and apps, allow users to continuously monitor their health status in real time—such as heart rate, activity levels, and sleep quality—and offer personalized health recommendations based on data analysis, thereby assisting users in achieving their health goals and alleviating CRF [20]. For patients experiencing mild fatigue symptoms, self-monitoring and adjustment of health behaviors using DHIs can effectively alleviate fatigue. For those with more severe symptoms, DHIs can offer more comprehensive personalized interventions and support, aiding in alleviating CRF symptoms.

### Objectives

Overall, DHIs can provide personalized, real-time, and flexible CRF management, which may more effectively address the challenges faced by adolescents and young adults in managing CRF, thereby enhancing treatment efficacy. However, the development of DHIs targeting CRF remains in its infancy, especially compared to those designed for other cancer-related conditions. In addition, some existing studies include adolescents and young adults and adults in mixed samples, and this may increase data heterogeneity [21]. Therefore, it is essential to synthesize the current evidence to assess the effectiveness of DHIs. A scoping review is especially useful for synthesizing evidence in emerging research fields. When the research question is unclear and specific hypotheses cannot be addressed through traditional systematic reviews, the scoping review method helps organize key concepts in the relevant research field, identify gaps in the existing literature, and systematically integrate emerging evidence [22]. To our knowledge, there have been no high-quality systematic or scoping reviews specifically focused on DHIs for CRF management in adolescents and young adults. This scoping review aims to summarize the current evidence on the types of DHIs for CRF management in adolescent and young adult patients and their effectiveness. In this context, this study holds dual significance. First, systematic reviews on the effectiveness of DHIs in CRF management within the adolescent and young adult group are insufficient, and this study aims to evaluate the specific effect of DHIs in CRF management within this group. Second, in light of the rapid advancements in digital health technologies, the adoption of a scoping review methodology effectively reduces the knowledge translation cycle, offering timely, evidence-based support to researchers and assisting clinicians in formulating empirically grounded CRF intervention strategies.

## Methods

We followed the 5-stage scoping review framework by Arksey and O’Malley [23] and the PRISMA-ScR (Preferred Reporting Items for Systematic Reviews and Meta-Analyses extension for Scoping Reviews) guidelines (Checklist 1) [24].

### Search Strategy

Researchers SJ and X Yang conducted a comprehensive search across 6 databases—PubMed, CINAHL, PsycINFO, Embase, Cochrane Library, and Web of Science—as well as a manual search of relevant references. The search spanned each database from its inception to August 2024 and was restricted to studies published in English. The search strategy involved a combination of MeSH (Medical Subject Heading) terms and keywords, including but not limited to (“digital health” OR “telemedicine”) AND (“adolescent” OR “young adult”) AND (“fatigue” OR “tired”) AND (“neoplasms” OR “carcinoma”)*.* Details of the search strategy are provided in Multimedia Appendix 1.

### Inclusion and Exclusion Criteria

Two researchers independently screened studies based on predefined inclusion and exclusion criteria. The inclusion criteria were as follows: (1) participants were patients with cancer or survivors aged 15 to 39 years, (2) the study used digital health technology as an intervention, (3) the study included fatigue-related outcomes, (4) the study was published in English, and (5) the full text was available. The exclusion criteria were reviews, protocols without results, case studies, reports, letters, conference proceedings, and abstract-only articles.

### Study Selection and Data Extraction

All relevant studies were imported into EndNote X9 (reference management software; Clarivate Analytics) to remove duplicates. Two researchers independently conducted an initial screening of titles and abstracts to evaluate whether they met the predetermined inclusion and exclusion criteria. Following this, they conducted a detailed full-text review to confirm each study’s eligibility. Any disagreements about study inclusion were resolved through consultation with a third researcher. The 2 authors independently extracted data from the included studies. They entered the data into a predesigned data extraction form that captured the following information: author, publication year, country, study design, participant ages, sample size, and cancer type. Any discrepancies were resolved through discussion or the involvement of a third researcher.

## Results

### Search Results

**Figure 1.**
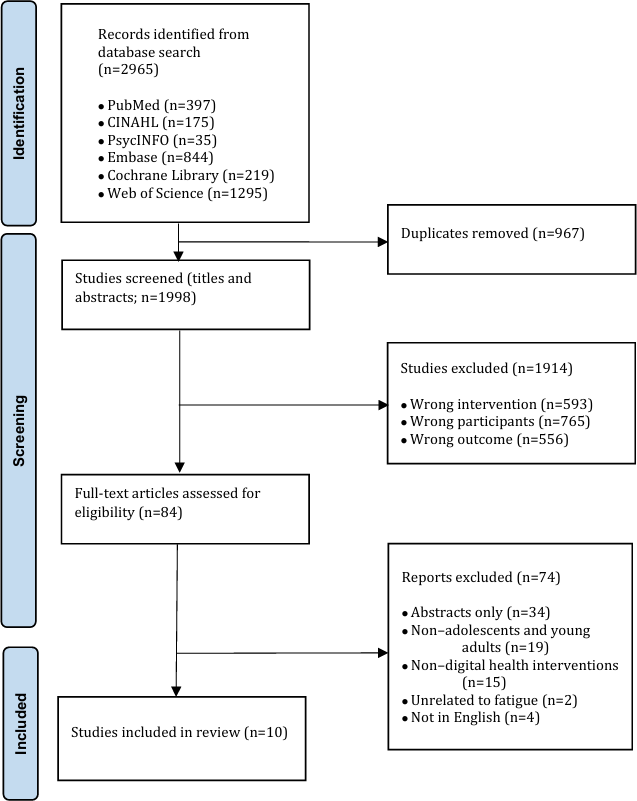
The PRISMA (Preferred Reporting Items for Systematic Reviews and Meta-Analyses) flow diagram.

A total of 2965 articles were identified through the literature search. Of these 2965 articles, after removing 967 (32.61%) duplicates, an additional 1914 (64.55%) were excluded based on title and abstract screening as they did not meet the inclusion criteria. This left 84 articles for full-text review, of which 74 (88%) were excluded due to inaccessible full texts, non-English language, incompatible study population, or unsuitable interventions. Ultimately, 10 studies were included in the data synthesis. Figure 1 shows the PRISMA (Preferred Reporting Items for Systematic Reviews and Meta-Analyses) flow diagram of the retrieved studies, the level of screening, and the included studies.

### Characteristics of the Selected Articles

The included studies were published in the years 2012 (1/10, 10%) [25], 2018 (1/10, 10%) [26], 2020 (2/10, 20%) [27,28], 2022 (1/10, 10%) [29], 2023 (3/10, 30%) [30-32], and 2024 (2/10, 20%) [33,34]. The 10 studies included 5 (50%) randomized controlled trials [25,28,29,31,33], 2 (20%) quasi-experimental studies [27,30], 2 (20%) mixed methods studies [32,34], and 1 (10%) cohort study [26]. Of the 10 studies, 5 (50%) were conducted in the United States [25-29]; 2 (20%) were conducted in Canada [32,33]; and 1 (10%) each was conducted in Australia [30], Turkey [31], and the Netherlands [34]. A detailed summary of the characteristics of the included studies is provided in Multimedia Appendix 2 [25-34].

### Summary of DHIs for Adolescents and Young Adults

#### Overview

Multimedia Appendix 2 provides a detailed description of the DHIs for adolescents and young adults. On the basis of the functions and forms of DHIs, we categorized them into the following 5 types: dynamic health monitoring and feedback (4/10, 40% of the studies) [26,28,29,33], automated online guidance and feedback (2/10, 20%) [25,27], live remote coaching and instruction (2/10, 20%) [32,34], gamified interventions (1/10, 10%) [31], and robot-assisted interventions (1/10, 10%) [30]. These DHIs were delivered through various technology platforms, including internet-based platforms (3/10, 30%), wearable devices with apps (3/10, 30%), wearable devices with internet-based programs (1/10, 10%), telehealth platforms (2/10, 20%), and robotic systems (1/10, 10%).

#### Dynamic Health Monitoring and Feedback

In total, 40% (4/10) of the studies explored the application of dynamic health monitoring and feedback technologies among adolescent and young adult patients with cancer. In this review, *dynamic health monitoring and feedback* refers to tracking and recording individual health data using wearable devices and assisting patients in adjusting their health behaviors through feedback provided by mobile apps.

In the study by Yurkiewicz et al [26], the intervention was based on Fitbit and iPad technology. Each participant received a Fitbit and an iPad Air preloaded with Headspace (a meditation app to support relaxation and mental health management), Re-Mission 2 (an interactive cancer-fighting game aimed at boosting morale), and the National Comprehensive Cancer Network Guidelines for Adolescent and Young Adult Oncology (informational sheets on treatment plans and recommendations to help participants understand their treatment). The Fitbit tracked steps, sleep, and calories burned. Participants could use these devices flexibly, either at the hospital or at home, and had access to an anonymous online community for social support. They were also allowed to download additional apps to suit their individual needs.

Devine et al [28] implemented an intervention called FitSurvivor grounded in social cognitive theory that emphasized the interactive influence of personal, environmental, and behavioral factors on individual behavior. The main objectives of the intervention were to enhance self-efficacy, goal setting, and self-monitoring; promote proper exercise posture; increase knowledge of cancer-related and general health behaviors; and provide social support. The intervention consisted of an initial 8-week face-to-face group course followed by a transition to a 4-week self-management program supported by a custom-built mobile app and Fitbit. Coaches assisted participants in setting specific exercise goals and provided weekly progress reviews. The app prompted participants to set goals upon their initial log-in and offered an exercise log feature, whereas the Fitbit enabled self-monitoring of steps and activity duration.

Tock et al [33] implemented the Lymfit intervention, which was designed based on self-determination theory, with the aim of enhancing patients’ motivation to engage in physical activity (PA) by fulfilling the needs of autonomy, competence, and relatedness. The Lymfit intervention was conducted remotely. Participants first received a Fitbit and resistance bands by mail and were guided through device setup via videoconference. All participants were then added to a virtual Lymfit lounge within the Fitbit app, which provided a peer support group. On the basis of baseline assessments, a kinesiologist developed a personalized 12-week exercise plan for each participant, with biweekly video follow-ups to adjust the plan and offer support. The Fitbit tracked daily PA and provided real-time feedback to assist participants in self-monitoring and maintaining motivation. This intervention combined personalized exercise guidance with virtual peer support, using regular plan adjustments to encourage sustained health management and improve participants’ exercise motivation and PA levels.

In the study conducted by Johnson et al [29], the intervention was similarly grounded in self-determination theory and was designed with a focus on the 3 core elements of autonomy, competence, and relatedness. The intervention included a wrist-worn PA tracking device (Fitbit), a Facebook group, step count goal setting, and a “buddy” system. Participants wore a Fitbit Flex activity tracker, set personalized goals, joined a Facebook group, participated in a PA “buddy” system, and received regular SMS text message reminders. The Fitbit tracked daily steps, and participants monitored their progress toward their goals through a mobile app. The Facebook group served as a social platform where participants could share their PA experiences, engage in discussions, and take part in group activities. In addition, participants could select an adult friend or family member as a “buddy” to provide encouragement and share exercise goals and achievements. They also received a daily motivational SMS text message related to PA at 3 PM Pacific Standard Time.

#### Automated Online Guidance and Feedback

A total of 20% (2/10) of the studies examined the use of automated online guidance and feedback for adolescent and young adult patients with cancer. In this review, *automated online guidance and feedback* refers to intervention services delivered through an internet platform. The system generates personalized health guidance and feedback automatically based on the patient’s individual information and needs.

In the study conducted by Rabin et al [25], the intervention was grounded in the transtheoretical model and social cognitive theory, facilitating PA by integrating the stages of behavior change with the interaction among the individual, behavior, and environment. Participants in the intervention group were given 12 weeks of access to the *Step Into Motion* website, where researchers guided them in setting weekly PA goals, logging activities, and completing questionnaires to generate personalized feedback. The website provided a customized PA manual, feedback reports, additional resources, a frequently asked question section, links to the benefits and precautions of PA for young survivors of cancer, and access to an online discussion forum.

In the study conducted by Zhou and Recklitis [27], the SHUTi-AYA automated intervention program was used. Grounded in cognitive behavioral therapy for insomnia, the program focuses on the interplay among cognition, emotions, and behaviors in the individual. It targets changing poor sleep habits, negative cognitive patterns, and emotional responses, thus improving sleep quality. SHUTi-AYA is an online, self-guided program that requires no external support. It offers personalized goal setting, graph-based feedback on insomnia symptoms, patient case studies, expert video explanations, and other interactive features, with content and visuals specifically tailored for adolescent and young adult survivors of cancer. After receiving personalized log-in credentials, participants completed 6 intervention sessions over an 8-week period, covering topics such as sleep restriction, stimulus control, cognitive therapy, sleep hygiene, and relapse prevention.

#### Live Remote Coaching and Instruction

In total, 20% (2/10) of the studies explored the use of live remote coaching and instruction among adolescent and young adult patients with cancer. In this review, *live remote coaching and instruction* refers to interactive courses and guidance delivered by professionals (eg, nurses, coaches, or therapists) via an online platform.

In the study by Wurz et al [32], the intervention was an 8-week online yoga program delivered via Zoom tailored for young people affected by cancer and their supporters. The program, developed from the evidence-based Yoga Thrive (a yoga program for patients with cancer) and the research team’s expertise, incorporated systematic reviews, virtual class preferences, and participant feedback. Each week, a 60-minute session was offered, including physical postures, breathing exercises, and meditation, complemented by self-guided resources, social support, and reflection prompts to encourage behavior change.

In the study conducted by Bouwman et al [34], the intervention, called REVIVER, combined cognitive behavioral therapy with motivational interviewing. It aimed to help participants identify and alter negative thought patterns, enhance self-awareness and motivation, and foster behavior change. The intervention was designed to assist child, adolescent, and young adult survivors of cancer in managing the direct or indirect late effects of cancer. The intervention sessions were facilitated by trained nurses via video coaching. Over 3 months, participants completed an initial interview and 3 to 6 video counseling sessions, followed by a reflection meeting 6 months later.

#### Gamified Interventions

A total of 10% (1/10) of the studies investigated the use of gamified interventions for adolescent and young adult patients with cancer. In this review, *gamified interventions* refer to interventions incorporating game elements into the health intervention process.

In the study by Uluhan and Akçay Didişen [31], participants used the Re-Mission video game, which was designed to enhance the physical and mental well-being of patients with cancer. The game aimed to increase adolescents’ understanding of the disease process, expand their knowledge of their condition, demonstrate potential physical changes during treatment, and enhance their understanding of both the effects and side effects of medication, all within a virtual environment. Participants received a game disc, with each session lasting 10 to 15 minutes, followed by a 1-hour break, with a maximum daily gameplay limit of 1 hour.

#### Robot-Assisted Interventions

A total of 10% (1/10) of the studies examined robot-assisted interventions among adolescent and young adult patients with cancer. In this review, *robot-assisted interventions* refer to those involving rehabilitation training conducted using robotic devices.

In the study by Atkinson et al [30], participants trained using either the Lokomat or ArmeoSpring rehabilitation robots focusing on gait or upper-limb function according to clinical needs, with only 1 type of training conducted per cycle. Training was conducted twice weekly over a 6-week period, starting with 20-minute sessions and gradually increasing to 40 minutes. Robot settings—such as reducing body weight support, increasing walking speed, decreasing guidance force, lowering upper-limb support, reducing motion control assistance, and increasing task speed—were customized based on participants’ abilities and goals. Trainers made progressive adjustments to the settings in line with guidelines from the American College of Sports Medicine and Exercise and Sports Science Australia. Heart rate and blood oxygen saturation were monitored during training using a Nellcor forehead peripheral oxygen saturation sensor, and training intensity was adjusted in response to participant feedback.

### CRF Outcomes

Of the 10 studies included in this review, 7 (70%) demonstrated a significant improvement in fatigue symptoms following the intervention. This section presents an analysis and summary of the results for each intervention type. Detailed data and further results are available in Multimedia Appendix 2.

In the dynamic health monitoring and feedback category, the study by Yurkiewicz et al [26] found a significant improvement in the *energy/fatigue* dimension of the RAND 36-Item Short Form Health Survey before and after the intervention, with scores increasing from 37.74 to 54.75 (*P*<.001). In the study by Devine et al [28], which used the Pediatric Quality of Life Inventory multidimensional fatigue scale, although the percentage increase generally favored the intervention group, there were no statistically significant group × time differences across fatigue outcomes after the intervention or at 6 months. In the study by Tock et al [33], the patient-reported outcomes measurement information system scale was used, and the results of the analysis of covariance model indicated a significant reduction in fatigue scores in the intervention group (*P*=.002). Johnson et al [29], through Fatigue Symptom Inventory scoring, found a significant difference in fatigue interference between the intervention and control groups (*P*=.03).

In the automated online guidance and feedback category, the study by Rabin et al [25] showed that the intervention group experienced a reduction in Profile of Mood States fatigue scores over 12 weeks. Although regression analysis did not reveal significant differences between the groups (*P*=.06), the findings suggested an alleviation of fatigue symptoms. Zhou and Recklitis [27] observed that, at 16 weeks after the intervention, participants’ scores on the Pediatric Quality of Life Inventory multidimensional fatigue scale were 70.9 (SD 22.2) on average, with an effect size of 1.2, corresponding to a large effect.

In the live remote coaching and instruction category, the study by Wurz et al [32] evaluated the improvement in fatigue symptoms using the Functional Assessment of Chronic Illness Therapy–Fatigue Scale. Although the *P* value was .10 and did not reach statistical significance, the effect size of 0.094 indicated a small to moderate improvement. Bouwman et al [34] assessed fatigue using the Checklist Individual Strength scale and found a significant reduction in fatigue symptoms after the intervention, with stable results maintained at the 6-month follow-up.

In the gamified intervention category, the study by Uluhan and Akçay Didişen [31] used the scale for the assessment of fatigue in pediatric oncology patients aged 13 to 18 years. After undergoing the Re-Mission video game intervention, the experimental group exhibited a significant reduction in fatigue scores at both the 1- and 3-month assessments compared to the control group (*P*<.001), indicating a significant improvement in fatigue level.

In the robot-assisted intervention category, the study by Atkinson et al [30] assessed fatigue using the Functional Assessment of Chronic Illness Therapy–Fatigue Scale. The results demonstrated a significant reduction in fatigue levels following Lokomat robotic training, with an effect size of *r*=0.53 and a *P* value of .04. For the ArmeoSpring robotic intervention, due to the limited sample size (n=3), only pre- and postintervention effect sizes were calculated, and inferential analysis was not conducted. However, preliminary findings suggested a large effect on fatigue improvement, with an effect size of *r*>0.5.

### Physical Function and Emotional Well-Being

A total of 9 studies reported outcomes related to physical function, of which 6 (67%) demonstrated significant improvements [26,27,29,31-34], whereas 3 (33%) did not show significant changes [25,28,30]. The studies that reported significant improvement involved the DHI categories of dynamic health monitoring and feedback, automated online guidance and feedback, live remote coaching and instruction, and gamified interventions. In contrast, the studies showing no significant improvement primarily focused on dynamic health monitoring and feedback, automated online guidance and feedback, and robot-assisted interventions. In terms of emotional well-being, 6 studies reported related outcomes, of which 4 (67%) demonstrated significant improvements [26,27,31,32], whereas 2 (33%) did not show significant changes [25,28]. The studies with significant improvements involved the DHI categories of dynamic health monitoring and feedback, automated online guidance and feedback, live remote coaching and instruction, and gamified interventions. The studies that showed no significant improvement primarily focused on dynamic health monitoring and feedback and automated online guidance and feedback.

## Discussion

### Principal Findings

This scoping review comprehensively examined the effectiveness of various DHIs in reducing CRF among adolescents and young adults, including dynamic health monitoring and feedback, automated online guidance and feedback, live remote coaching and instruction, gamified interventions, and robot-assisted interventions. Multiple studies demonstrated significant changes in participants’ fatigue scores following the interventions, with effects that persisted beyond the intervention period, indicating that DHIs are notably effective in alleviating CRF. In addition, DHIs also demonstrated potential in improving physical function and emotional health in this population. DHIs are well suited to the behavioral characteristics and preferences of adolescents and young adults, offering flexible, multidimensional support for CRF management and highlighting distinct advantages in this population.

*Dynamic health monitoring and feedback* refers to the use of apps and wearable devices to collect health data in real time and provide feedback. This enables patients to promptly identify potential health concerns and dynamically adjust their lifestyle and rehabilitation plans based on data-driven feedback [35,36]. For adolescents and young adults, the strength of these programs lies in their capacity to offer comprehensive support and high flexibility, aligning with their daily life rhythms. They can be easily incorporated into school, work, or social activities. Wearable devices continuously collect health data, whereas the apps process and analyze these data. This dynamic health monitoring and feedback assists adolescents and young adults in effectively managing their health through features such as setting personalized goals, tracking progress, sending regular reminders, and facilitating social interactions. This approach enables adolescents and young adults to track health changes continuously amid their busy lives and receive emotional support and encouragement, thus enhancing self-management motivation; improving exercise adherence; fostering healthy behavior habits; and, ultimately, alleviating CRF symptoms.

However, some studies did not show improvements in CRF [28,29]. This may be due to factors such as the limitations of the intervention content, insufficient intervention design, and underuse of social support. First, some intervention programs primarily focused on strength training and health education, but improving fatigue typically requires a more comprehensive approach [37]. For instance, incorporating aerobic training and psychological support can better address both the physiological and psychological aspects of fatigue, thereby enhancing the effectiveness of the intervention. Second, in interventions involving reminder systems, excessively frequent reminders may cause participants to focus too much on their fatigue, increasing their sensitivity and amplifying its negative impact on quality of life. In addition, although some programs included a social support component, the underuse of this support by participants led to suboptimal outcomes in this area, weakening the overall potential of the intervention to alleviate fatigue [35]. Therefore, future dynamic health monitoring and feedback programs should optimize their content by integrating more aerobic training and psychological support to comprehensively target the physiological and psychological factors of fatigue. Furthermore, reminder systems should be personalized to avoid the negative effects of excessive reminders. The design of social support modules should also be refined to enhance the quality of social interactions, thereby improving participant engagement and the sustainability of the intervention, and more effectively alleviating CRF symptoms in adolescents and young adults.

Automated online guidance and feedback interventions provide adolescents and young adults with personalized, automated health guidance via web platforms or applications. Using algorithms and preset programs, these interventions automatically generate health advice and feedback based on participants’ input data, ensuring that continuous support is accessible anytime and anywhere [36,38]. For busy adolescents and young adults, automated systems can rapidly respond to their needs, delivering timely and personalized recommendations through data-driven feedback, thereby reducing the delays typically associated with manual interventions. This supports participants in adhering more effectively to their rehabilitation plans, thereby facilitating effective CRF management and improving overall health management outcomes. Regarding interventions that did not lead to significant improvements, a potential reason could be that participants were unable to fully engage due to time constraints, which impacted the overall effectiveness of fatigue management [25]. To address this, automated online guidance and feedback interventions could incorporate the concept of microexercises. This approach would enable participants to perform short, efficient sessions within their busy schedules, thereby overcoming the common barrier of time constraints associated with longer workouts. In addition, the platform could integrate features such as health tracking, dietary advice, and stress management tools, further supporting comprehensive fatigue management. This would ensure that participants can maintain continuous fatigue management even when extended exercise sessions are not possible.

Live remote coaching and instruction interventions provide personalized health guidance and support to adolescents and young adults through online tools such as videoconferencing. A key advantage of these interventions is their ability to overcome geographical and temporal constraints, making them especially suitable for this population’s complex schedules and varied lifestyle needs. This model allows participants to receive high-quality guidance without the need to travel to specific locations, significantly enhancing the intervention’s convenience and flexibility and facilitating the smoother integration of rehabilitation into daily life [39]. In addition, live remote coaching and instruction offers adolescents and young adults the option to conduct rehabilitation at home or in other familiar settings, effectively reducing psychological stress and anxiety. This is particularly beneficial for managing CRF in adolescents and young adults as it minimizes the added fatigue associated with travel or unfamiliar environments, ultimately optimizing rehabilitation outcomes [40].

Gamified interventions effectively enhance the motivation and engagement of adolescents and young adults through interactive games. Given that this age group is particularly drawn to novel and engaging activities, the interactive and enjoyable nature of games can stimulate their motivation for PA, alleviating fatigue through enjoyable experiences and positive feedback, thereby optimizing overall fatigue management [41]. A key advantage of gamified interventions lies in their ability to integrate healthy behaviors within the game context, allowing participants to engage in health-related activities in a natural manner. In-game challenges and achievement systems provide adolescents and young adults with a sense of progress and accomplishment, which plays a crucial role in alleviating fatigue. Furthermore, gamification elements help structure health activities, enabling participants to consistently follow a well-organized plan, leading to more effective fatigue management [42].

Robot-assisted interventions provide more stable and reliable treatment than human-assisted methods through precise control and dynamically adjustable intensity. This high level of control accurately simulates natural body movements, ensuring optimal effectiveness in each session and allowing for personalized adjustments based on individual needs, thus enhancing both effectiveness and adaptability [43]. Moreover, robot-assisted technology is highly interactive and appeals to adolescents and young adults, who tend to show strong interest in emerging technologies and interactive experiences. The advanced, high-technology aspect of robot-assisted interventions can boost adolescents and young adults’ motivation to participate, making the intervention process more engaging and sustainable. This technology-driven experience helps robot-assisted technology integrate more effectively into the treatment process, facilitating better management of CRF and improving intervention outcomes.

In the studies included in this review, those that did not yield significant results predominantly involved posttreatment patients with cancer. Thus, the effectiveness of DHIs in managing CRF among patients with cancer may be influenced by the treatment phase, and the selection of the type of DHI should be adaptable depending on the treatment phase. During the course of treatment, patients frequently experience severe fatigue directly associated with the treatment [44]. As a result, dynamic health monitoring and feedback–based DHIs are especially crucial. These technologies enable real-time monitoring of the patient’s physical condition (eg, heart rate, sleep, and activity levels) and provide immediate feedback, assisting patients in better managing treatment-related discomfort. Furthermore, gamified interventions are highly appropriate for patients during treatment as their engaging and interactive characteristics can capture patients’ interest and motivate them to participate more actively in PAs.

However, during the later phase of treatment, particularly in the recovery phase following cancer treatment, patients’ needs evolve [45]. At this stage, fatigue typically becomes a chronic and persistent issue, making automated online guidance and feedback or live remote coaching and instruction–type DHIs more appropriate. These technologies allow patients to receive personalized health recommendations and interventions tailored to their symptoms, helping them learn how to manage fatigue in daily life, restore physical strength, and make adjustments to their diet and lifestyle while alleviating the difficulties arising from limited medical resources or the inconvenience of accessing care during treatment. In addition, gamified interventions can play a positive role in the later phase of treatment. By promoting patients’ adherence to PA through engaging interventions, these approaches help alleviate fatigue.

For patients with severe CRF who need long-term rehabilitation, robot-assisted rehabilitation offers an effective intervention. Robotic technology enables patients to undergo personalized physical rehabilitation training, enhancing physical functions such as balance, coordination, and muscle strength; restoring motor function [46]; and alleviating CRF symptoms.

Furthermore, research has indicated that DHIs show potential in improving both the physical function and emotional well-being of adolescent and young adult patients with cancer. These DHIs promote exercise adherence and motivation among adolescent and young adult patients; enhance their PA levels; and improve physical function through engaging exercise approaches, personalized exercise protocols, and real-time monitoring feedback. However, some studies did not show significant improvements in physical function, which may be attributed to the nature of the intervention technology and its usability experience. Research has shown that patients’ active participation and self-efficacy are strongly associated with improvements in physical function [47]. However, the design of certain DHIs may have inadequately considered patient interaction, resulting in insufficient active patient participation, which in turn impairs the effectiveness of the intervention. Therefore, future DHIs should focus on the design of interactive modules to promote patients’ autonomy and engagement, thus enhancing the effectiveness of the intervention. With respect to emotional well-being, DHIs help patients manage psychological stress during treatment by providing virtual social support and personalized guidance and interaction, thereby reducing anxiety and depression and fostering mental health recovery. However, some studies did not demonstrate significant emotional improvements, which may result from these interventions primarily focusing on physical health and behavior changes while the emotional support component remains relatively underdeveloped. When emotional support is insufficiently prioritized, the intensity of the intervention may be inadequate, leading to patients lacking continuous emotional support, which in turn impedes improvements in emotional well-being. Therefore, future DHIs should enhance the emotional support component to ensure that they not only address physical health but also provide robust emotional support and psychological guidance, thus amplifying the intervention’s positive effects on emotional well-being.

In summary, DHIs that integrate digital health technologies with various intervention forms have demonstrated significant benefits in managing CRF in the adolescent and young adult population. Digital tools, due to their flexibility, personalization, engaging nature, and interactivity, not only transcend the limitations of time and space to cater to the diverse needs of adolescent and young adult patients across various living environments but also more effectively enhance CRF management by fostering greater patient engagement and long-term adherence. Simultaneously, by incorporating various intervention strategies, including behavioral interventions, emotional regulation, social interaction, and exercise motivation, they offer a comprehensive and integrated support system. This comprehensive intervention model transcends the time and spatial limitations of traditional treatments and optimizes CRF management by integrating multidimensional interventions that encompass physical, psychological, and social aspects. This highlights the significant potential of combining digital health technologies with multidimensional interventions, offering new perspectives on managing cancer-related symptoms and furthering the application of DHIs in symptom management.

### Future Research

Although this review discussed the potential effects of various DHIs in reducing CRF among adolescents and young adults, several unresolved questions require further investigation. First, future research should prioritize larger sample sizes and use more rigorous study designs such as randomized controlled trials to enhance the external validity and statistical significance of the findings. Second, studies should explore the long-term effects of DHIs. Currently, most research assesses only short-term outcomes, whereas CRF, as a chronic symptom, requires sustained management to ensure optimal patient quality of life and health outcomes. Therefore, long-term follow-up studies are needed to evaluate the lasting effects of these interventions on fatigue reduction, quality of life, and recovery. In addition, the studies included in this review used various scales for fatigue assessment, underscoring the multidimensional nature of fatigue as a symptom. Future research may need to explore integrating multiple scales to capture the full spectrum of fatigue.

### Limitations

The studies included in this review involved a diverse range of cancer types, with large variability in sample sizes across cancer types, thus preventing exploration of the relationship between specific cancer types and the choice of DHIs. In addition, this review included only 10 studies, of which 5 (50%) had small sample sizes, which may affect the generalizability of the results. Moreover, while the scoping review approach provides a broad overview, it does not assess the quality of included studies, which may affect the overall strength of the evidence.

### Conclusions

This scoping review highlights the positive impact of DHIs in alleviating CRF among adolescents and young adults. Through diverse technological methods, DHIs offer a more engaging and interactive approach, effectively enhancing adherence and self-management skills in adolescent and young adult patients while also facilitating access to psychological and social support. DHIs demonstrate significant potential for delivering personalized, patient-centered cancer care, providing adolescent and young adult patients with cancer with new avenues to manage CRF and offering compelling evidence for the role of DHIs in symptom management.

## Supplementary material

10.2196/68834 Multimedia Appendix 1 Search strategy.

10.2196/68834 Multimedia Appendix 2 Description of digital health interventions and related outcomes.

10.2196/68834 Checklist 1 PRISMA-ScR checklist.

## Abbreviations

CRF: cancer-related fatigue
DHI: digital health intervention
MeSH: Medical Subject Heading
PA: physical activity
PRISMA: Preferred Reporting Items for Systematic Reviews and Meta-Analyses
PRISMA-ScR: Preferred Reporting Items for Systematic Reviews and Meta-Analyses Extension for Scoping Reviews
