# Supplementary material for: Digital Health Interventions to Reduce Cancer-Related Fatigue Among Adolescents and Young Adults: Scoping Review
Source: JMIR Mhealth Uhealth. 2025 Oct 21;13:e68834. doi: 10.2196/68834 (PMC12539328; doi:10.2196/68834)
Supplement: Multimedia Appendix 1 [file mhealth-v13-e68834-s001.docx]

Multimedia Appendix 1. search strategy

| 1. Pubmed | |
| --- | --- |
| #1 | Telemedicine[MeSH Terms] OR “Digital Health” [MeSH Terms] |
| #2 | telenursing[Title/Abstract] OR telecare[Title/Abstract] OR telecommunicat*[Title/Abstract] OR telepalliative[Title/Abstract] OR “remote consult*”[Title/Abstract] OR telemonitor*[Title/Abstract] OR teleconsult*[Title/Abstract] OR telehealth*[Title/Abstract] OR app[Title/Abstract] OR apps[Title/Abstract] OR application[Title/Abstract] OR “virtual medicine”[Title/Abstract] OR e-health[Title/Abstract] OR m-health[Title/Abstract] OR mhealth[Title/Abstract] OR eHealth[Title/Abstract] OR mobile[Title/Abstract] OR cellphone[Title/Abstract] OR online[Title/Abstract] OR internet[Title/Abstract] OR website[Title/Abstract] OR smartphone[Title/Abstract] OR web-based[Title/Abstract] OR Video[Title/Abstract] OR technolog*[Title/Abstract] OR digital*[Title/Abstract] OR electronic*[Title/Abstract] OR game[Title/Abstract] OR message[Title/Abstract] OR “Nintendo Wii”[Title/Abstract] OR VR[Title/Abstract] OR gamif*[Title/Abstract] OR “social media”[Title/Abstract] OR we-chat[Title/Abstract] OR tracker*[Title/Abstract] |
| #3 | #1 OR #2 |
| #4 | Adolescent[MeSH Terms] OR Young Adult [MeSH Terms] |
| #5 | adolescen*[Title/Abstract] OR minor*[Title/Abstract] OR puberty[Title/Abstract] OR juvenile*[Title/Abstract] OR teen*[Title/Abstract] OR “young adult*” [Title/Abstract] OR youth*[Title/Abstract] OR AYA [Title/Abstract] OR AYAs[Title/Abstract] |
| #6 | #4 OR #5 |
| #7 | Fatigue[MeSH Terms] |
| #8 | fatigue[Title/Abstract] OR tired*[Title/Abstract] OR exhaust*[Title/Abstract] OR asthenia [Title/Abstract] OR lassitude[Title/Abstract] OR lackluster[Title/Abstract] OR letharg*[Title/Abstract] OR “wear* out”[Title/Abstract] OR “cancer-related fatigue”[Title/Abstract] OR “cancer related fatigue”[Title/Abstract] |
| #9 | #7 OR #8 |
| #10 | Neoplasms[MeSH Terms] OR Carcinoma[MeSH Terms] |
| #11 | cancer[Title/Abstract] OR oncology[Title/Abstract] OR tumor*[Title/Abstract] OR tumour*[Title/Abstract] OR malignan*[Title/Abstract] OR neoplas*[Title/Abstract] OR carcinoma*[Title/Abstract] OR metasta*[Title/Abstract] OR leukemi*[Title/Abstract] |
| #12 | #10 OR #11 |
| #13 | #3 AND #6 AND #9 AND #12 |
| 2. Cochrane Library | |
| #1 | MeSH descriptor: [Telemedicine] explode all trees |
| #2 | MeSH descriptor: [Digital Health] explode all trees |
| #3 | #1 OR #2 |
| #4 | (telenursing OR telecare OR telecommunicat* OR telepalliative OR “remote consult” OR telemonitor* OR teleconsult* OR telehealth* OR app OR apps OR application OR “virtual medicine” OR e-health OR m-health OR mhealth OR eHealth OR mobile OR cellphone OR online OR internet OR website OR smartphone OR web-based OR Video OR technolog* OR digital* OR electronic* OR game OR message OR “Nintendo Wii” OR VR OR gamif* OR “social media” OR we-chat OR tracker*):ti,ab,kw |
| #5 | #3 OR #4 |
| #6 | MeSH descriptor: [Adolescent] explode all trees |
| #7 | MeSH descriptor: [Young Adult] explode all trees |
| #8 | #6 OR #7 |
| #9 | (adolescen* OR minor* OR puberty OR juvenile* OR teen* OR “young adult” OR “young adults” OR youth* OR AYA*):ti,ab,kw |
| #10 | #8 OR #9 |
| #11 | MeSH descriptor: [Fatigue] explode all trees |
| #12 | (fatigue OR tired* OR exhaust* OR asthenia OR lassitude OR lackluster OR letharg* OR “wear out” OR “cancer-related fatigue” OR “cancer related fatigue”):ti,ab,kw |
| #13 | #11 OR #12 |
| #14 | MeSH descriptor: [Neoplasms] explode all trees |
| #15 | MeSH descriptor: [Carcinoma] explode all trees |
| #16 | #14 OR #15 |
| #17 | (cancer OR oncology OR tumor* OR tumour* OR malignan* OR neoplas* OR carcinoma* OR metasta* OR leukemi*):ti,ab,kw |
| #18 | #16 OR #17 |
| #19 | #5 AND #10 AND #13 AND #18 |
| 3. Web of Science | |
| #1 | TS=(telenursing OR telecare OR telecommunicat* OR telepalliative OR “remote consult*” OR telemonitor* OR teleconsult* OR telehealth* OR app OR apps OR application OR “virtual medicine” OR e-health OR m-health OR mhealth OR eHealth OR mobile OR cellphone OR online OR internet OR website OR smartphone OR web-based OR Video OR technolog* OR digital* OR electronic* OR game OR message OR “Nintendo Wii” OR VR OR gamif* OR “social media” OR we-chat OR tracker*) |
| #2 | TS=( adolescen* OR minor* OR puberty OR juvenile* OR teen* OR “young adult” OR “young adults” OR youth* OR AYA*) |
| #3 | TS=( fatigue OR tired* OR exhaust* OR asthenia OR lassitude OR lackluster OR letharg* OR “wear* out” OR “cancer-related fatigue” OR “cancer related fatigue”) |
| #4 | TS=(cancer OR oncology OR tumor* OR tumour* OR malignan* OR neoplas* OR carcinoma* OR metasta* OR leukemi*) |
| #5 | #1 AND #2 AND #3 AND #4 |
| 4. Embase | |
| #1 | 'telemedicine'/exp OR 'digital health'/exp |
| #2 | telenursing:ti,ab,kw OR telecare:ti,ab,kw OR telecommunicat*:ti,ab,kw OR telepalliative:ti,ab,kw OR 'remote consult*':ti,ab,kw OR telemonitor*:ti,ab,kw OR teleconsult*:ti,ab,kw OR telehealth*:ti,ab,kw OR app:ti,ab,kw OR apps:ti,ab,kw OR application:ti,ab,kw OR 'virtual medicine':ti,ab,kw OR 'e health':ti,ab,kw OR 'm health':ti,ab,kw OR mhealth:ti,ab,kw OR ehealth:ti,ab,kw OR mobile:ti,ab,kw OR cellphone:ti,ab,kw OR online:ti,ab,kw OR internet:ti,ab,kw OR website:ti,ab,kw OR smartphone:ti,ab,kw OR 'web based':ti,ab,kw OR video:ti,ab,kw OR technolog*:ti,ab,kw OR digital*:ti,ab,kw OR electronic*:ti,ab,kw OR game:ti,ab,kw OR message:ti,ab,kw OR 'nintendo wii':ti,ab,kw OR vr:ti,ab,kw OR gamif*:ti,ab,kw OR 'social media':ti,ab,kw OR 'we chat':ti,ab,kw OR tracker*:ti,ab,kw |
| #3 | #1 OR #2 |
| #4 | 'adolescent'/exp OR 'young adult'/exp |
| #5 | adolescen*:ti,ab,kw OR minor*:ti,ab,kw OR puberty:ti,ab,kw OR juvenile*:ti,ab,kw OR teen*:ti,ab,kw OR 'young adult':ti,ab,kw OR 'young adults':ti,ab,kw OR youth*:ti,ab,kw OR AYA*:ti,ab,kw |
| #6 | #4 OR #5 |
| #7 | 'fatigue'/exp |
| #8 | fatigue:ti,ab,kw OR tired*:ti,ab,kw OR exhaust*:ti,ab,kw OR asthenia:ti,ab,kw OR lassitude:ti,ab,kw OR lackluster:ti,ab,kw OR letharg*:ti,ab,kw OR 'wear* out':ti,ab,kw OR 'cancer-related fatigue':ti,ab,kw OR 'cancer related fatigue':ti,ab,kw |
| #9 | #7 OR #8 |
| #10 | 'neoplasm'/exp OR 'carcinoma'/exp |
| #11 | cancer:ti,ab,kw OR oncology:ti,ab,kw OR tumor*:ti,ab,kw OR tumour*:ti,ab,kw OR malignan*:ti,ab,kw OR neoplas*:ti,ab,kw OR carcinoma*:ti,ab,kw OR metasta*:ti,ab,kw OR leukemi*:ti,ab,kw |
| #12 | #10 OR #11 |
| #16 | #3 AND #6 AND #9 AND #12 |
| 5. PsycINFO | |
| S1 | DE "Telemedicine" |
| S2 | TI (telenursing OR telecare OR telecommunicat* OR telepalliative OR “remote consult*” OR telemonitor* OR teleconsult* OR telehealth* OR app OR apps OR application OR “virtual medicine” OR e-health OR m-health OR mhealth OR eHealth OR mobile OR cellphone OR online OR internet OR website OR smartphone OR web-based OR Video OR technolog* OR digital* OR electronic* OR game OR message OR “Nintendo Wii” OR VR OR gamif* OR “social media” OR we-chat OR tracker*) OR AB (telenursing OR telecare OR telecommunicat* OR telepalliative OR “remote consult*” OR telemonitor* OR teleconsult* OR telehealth* OR app OR apps OR application OR “virtual medicine” OR e-health OR m-health OR mhealth OR eHealth OR mobile OR cellphone OR online OR internet OR website OR smartphone OR web-based OR Video OR technolog* OR digital* OR electronic* OR game OR message OR “Nintendo Wii” OR VR OR gamif* OR “social media” OR we-chat OR tracker*) |
| S3 | S1 OR S2 |
| S4 | DE " Emerging Adulthood" |
| S5 | TI (adolescen* OR minor* OR puberty OR juvenile* OR teen* OR “young adult” OR “young adults” OR youth* OR AYA*) OR AB (adolescen* OR minor* OR puberty OR juvenile* OR teen* OR “young adult” OR “young adults” OR youth* OR AYA*) |
| S6 | S4 OR S5 |
| S7 | DE " Fatigue" |
| S8 | TI (fatigue OR tired* OR exhaust* OR asthenia OR lassitude OR lackluster OR letharg* OR “wear* out” OR “cancer-related fatigue” OR “cancer related fatigue”) OR AB (fatigue OR tired* OR exhaust* OR asthenia OR lassitude OR lackluster OR letharg* OR “wear* out” OR “cancer-related fatigue” OR “cancer related fatigue”) |
| S9 | S7 OR S8 |
| S10 | DE "Neoplasms" |
| S11 | TI (cancer OR oncology OR tumor* OR tumour* OR malignan* OR neoplas* OR carcinoma* OR metasta* OR leukemi*) OR AB (cancer OR oncology OR tumor* OR tumour* OR malignan* OR neoplas* OR carcinoma* OR metasta* OR leukemi*) |
| S12 | S10 OR S11 |
| S16 | S3 AND S6 AND S9 AND S12 |
| 6. CINAHL | |
| S1 | (MH "Telemedicine+") OR (MH "Digital Health+") |
| S2 | TI (telenursing OR telecare OR telecommunicat* OR telepalliative OR “remote consult*” OR telemonitor* OR teleconsult* OR telehealth* OR app OR apps OR application OR “virtual medicine” OR e-health OR m-health OR mhealth OR eHealth OR mobile OR cellphone OR online OR internet OR website OR smartphone OR web-based OR Video OR technolog* OR digital* OR electronic* OR game OR message OR “Nintendo Wii” OR VR OR gamif* OR “social media” OR we-chat OR tracker*) OR AB (telenursing OR telecare OR telecommunicat* OR telepalliative OR “remote consult*” OR telemonitor* OR teleconsult* OR telehealth* OR app OR apps OR application OR “virtual medicine” OR e-health OR m-health OR mhealth OR eHealth OR mobile OR cellphone OR online OR internet OR website OR smartphone OR web-based OR Video OR technolog* OR digital* OR electronic* OR game OR message OR “Nintendo Wii” OR VR OR gamif* OR “social media” OR we-chat OR tracker*) |
| S3 | S1 OR S2 |
| S4 | (MH "Adolescence+") OR (MH "Young Adult") |
| S5 | TI (adolescen* OR minor* OR puberty OR juvenile* OR teen* OR “young adult” OR “young adults” OR youth* OR AYA*) OR AB (adolescen* OR minor* OR puberty OR juvenile* OR teen* OR “young adult” OR “young adults” OR youth* OR AYA*) |
| S6 | S4 OR S5 |
| S7 | (MH "Fatigue+") OR (MH "Cancer Fatigue+") |
| S8 | TI (fatigue OR tired* OR exhaust* OR asthenia OR lassitude OR lackluster OR letharg* OR “wear* out” OR “cancer-related fatigue” OR “cancer related fatigue”) OR AB (fatigue OR tired* OR exhaust* OR asthenia OR lassitude OR lackluster OR letharg* OR “wear* out” OR “cancer-related fatigue” OR “cancer related fatigue”) |
| S9 | S7 OR S8 |
| S10 | (MH "Neoplasms+") OR (MH "Carcinoma+") |
| S11 | TI (cancer OR oncology OR tumor* OR tumour* OR malignan* OR neoplas* OR carcinoma* OR metasta* OR leukemi*) OR AB (cancer OR oncology OR tumor* OR tumour* OR malignan* OR neoplas* OR carcinoma* OR metasta* OR leukemi*) |
| S12 | S10 OR S11 |
| S13 | S3 AND S6 AND S9 AND S12 |
