# Supplementary material for: Digital Health Interventions to Reduce Cancer-Related Fatigue Among Adolescents and Young Adults: Scoping Review
Source: JMIR Mhealth Uhealth. 2025 Oct 21;13:e68834. doi: 10.2196/68834 (PMC12539328; doi:10.2196/68834)
Supplement: Multimedia Appendix 2 [file mhealth-v13-e68834-s002.docx]

| Multimedia Appendix 2. Description of digital health interventions (DHIs) and related outcomes. | | | | | | | | | | | | | | | |  |
| --- | --- | --- | --- | --- | --- | --- | --- | --- | --- | --- | --- | --- | --- | --- | --- | --- |
| Types of DHI | | Author year | Country | Study design | Age Range (years) | N | Type of cancer | Treatment phase | Theoretical framework | Names of DHI | Platforms for DHI | Content of DHI | Duration | Main outcome measure | Main outcome | Secondary outcomes |
| **Dynamic Health Monitoring and Feedback** | |  |  |  |  |  |  |  |  |  |  |  |  |  |  |  |
|  | | Yurkiewicz 2018 [25] | America | cohort study | 15–28 | 33 | Various types (Testicular, Thyroid, Leukemia or Lymphoma, Breast, Other) | New Diagnosis | \ | Fitbit and iPad Technology | Wearable Devices with App | Participants used iPads preloaded with well-being apps, including Headspace (for meditation), Remission 2 (a cancer-fighting game), and the NCCN AYA guidelines (for treatment information), along with Fitbits that tracked steps, sleep, and calories. | one year | RAND-36^a^ | The mean score on the “Energy/Fatigue” dimension increased from 37.74 before the intervention to 54.75 after the intervention, with a *P*-value of < .001. | Physical function, emotional well-being |
|  | | Devine 2020 [27] | America | RCT | 13–25 | 49 | Blood cancer, Brain tumor, Solid tumor | Discontinue treatment for ≥6 months | social cognitive theory | FitSurvivor | Wearable Devices with App | Eight weeks of in-person group sessions were followed by four weeks of app and Fitbit use. Coaches assisted participants in setting specific exercise goals and conducted weekly progress reviews. The app prompted users to set goals upon first login and included an exercise log feature, while the Fitbit was used for self-monitoring steps and activity duration. | 12 weeks | PedsQL multidimensional fatigue scale^b^ | Although the percent increase generally favored the intervention group, there were no statistically significant group × time differences across fatigue outcomes post-intervention or at 6 months. | Physical function, emotional well-being |
|  | | Tock 2024[32] | Canada | RCT | 20–39 | 26 | Lymphoma | Undergoing chemotherapy or completed chemotherapy in the past 6 months | Self-determination theory (SDT) | Lymfit | Wearable Devices with App | Participants used a pre-assigned Fitbit to track their activity, received individualized exercise programs based on baseline assessments, and attended bi-weekly follow-up sessions over 12 weeks to review and adjust their progress. | 12 weeks | PROPr^c^ | The ANCOVA model results showed a significant reduction in the fatigue score for the intervention group post-intervention, with an effect size (ES) of 0.346, indicating a moderate effect. The minimal clinically important difference (MCID) analysis revealed a decrease of 7.36 in the fatigue score for the intervention group, surpassing the MCID threshold. | Physical function |
|  | | Johnson 2022 [28] | America | RCT | 18–39 | 49 | Various types (Brain, Breast, Lymphoma, Leukemia, Other) | Cancer treatment completed ≥ 1 year and < 5 years ago | Self-determination theory (SDT) | Fitbit- and Facebook-Based Physical Activity | Wearable Devices with Internet | The intervention consisted of using a Fitbit Flex activity tracker, setting personalized goals, joining a Facebook group, participating in a PA "buddy" system for support, and receiving regular text message reminders to encourage engagement. | 12 weeks | FSI 15-item^d^ | The fatigue interference score in the intervention group increased from 2.3 (95% CI 1.5–3.0) to 3.2 (95% CI 2.4–3.9), while the score in the control group remained at 2.4 (95% CI 1.6–3.2) at both baseline and follow-up. A significant difference was observed in the changes in fatigue interference scores between the two groups, with a *P*-value of .03. | Physical function |
| **Automated Online Guidance and Feedback** | |  |  |  |  |  |  |  |  |  |  |  |  |  |  |  |
|  | | Rabin 2012 [24] | America | RCT | 18–39 | 18 | Various types (Testicular, Thyroid, Leukemia or Lymphoma, Breast, Other) | All treatments completed, in cancer remission | Transtheoretical model, social cognitive theory | Customized Physical Activity Website | Internet | Participants were granted access to the "Step into Motion (SIM)" website, where researchers assisted them in setting weekly physical activity (PA) goals, tracking their progress, and completing questionnaires to receive personalized feedback. The website provided individualized PA guides, feedback reports, relevant resources, and an online discussion forum to support participants in planning and managing their PA effectively. | 12 weeks | POMS^e^ | The fatigue scores in the intervention group decreased from mean 18.71 (SD 13.66) to mean 7.28 (SD 7.61) over the 12-week period, representing a mean reduction of mean 11.43 points (SD 11.65). However, the difference between the groups was not statistically significant (*P*=.06). | Physical function, emotional well-being |
|  | | Zhou 2020[26] | America | Quasi-experimental study | 14–25 | 22 | Blood cancer, Solid tumor | Discontinue treatment for ≥3 months | Cognitive-behavioral therapy for insomnia (CBT-I) | SHUTi-AYA | Internet | The intervention was delivered through SHUTi-AYA, an automated online program based on Cognitive Behavioral Therapy for Insomnia (CBT-I), specifically designed for AYA cancer survivors. Over the course of 8 weeks, participants completed six intervention sessions. | 6—8 weeks | PedsQL multidimensional fatigue scale^b^ | The scores on the PedsQL multidimensional fatigue scale were mean 46.1 (SD 16.7) at baseline and increased to mean 63.2 (SD 22.5) at 8 weeks post-intervention, with an effect size of 0.7, indicating a moderate effect. At 16 weeks post-intervention, the fatigue score further increased to mean 70.9 (SD 22.2), with an effect size of 1.2, reflecting a large effect. | Physical function, emotional well-being |
| **Live Remote Coaching and Instruction** | |  |  |  |  |  |  |  |  |  |  |  |  |  |  |  |
|  | | Wurz 2023[31] | Canada | mixed methods study | 18–39 | 30 | Various types (Blood, Breast, Digestive, genitourinary, Gynecological, Head and neck, lung, Metastases, Neurological, Skin, Thyroid, Other) | Any stage of cancer progression (i.e., from the initial diagnosis) | \ | yoga intervention delivered by video conference | App | The intervention was an online yoga program delivered via Zoom, specifically designed for young people affected by cancer and their supporters. Each week, a 60-minute session was offered, which included physical poses, breathing exercises, and meditation, complemented by self-guided support, social support, and reflection prompts. | 8 weeks | FACIT-F^f^ | In the FACIT-Fatigue scale, the baseline score was 30.73, rising to 32.50 after the intervention and 33.81 at follow-up. Although the *P*-value of .097 did not reach statistical significance, the effect size was 0.094, suggesting a small to moderate improvement. | Physical function, emotional well-being |
|  | | Bouwman 2024 [33] | Netherlands | mixed methods study | 16–54 | 35 | Various types (Leukemia, Hodgkin lymphoma, Non-Hodgkin lymphoma, CNS tumors, Neuroblastoma, Renal tumors, Germ cell tumors, Bone tumors, Soft tissue tumors, Other) | Cancer remission | cognitive behavior therapy (CBT), motivational interviewing (MI) | REVIVER | App | Participants participated in an initial interview and 3 to 6 video coaching sessions over the course of three months, followed by a reflection meeting at six months. The intervention was delivered by trained nurses and incorporated Cognitive Behavioral Therapy (CBT), Motivational Interviewing (MI), or a combination of both. | 6 months | CIS20^g^ | From baseline (T0) to post-intervention (T1), both overall fatigue and fatigue severity scores significantly decreased (*d*=-1.34 and *d*=-1.69, respectively), with improvements sustained at the 6-month follow-up (T2). | Physical function |
| **Gamified Intervention** | |  |  |  |  |  |  |  |  |  |  |  |  |  |  |  |
|  | | Uluhan 2023 [30] | Turkey | RCT | 13–17 | 46 | Acute leukemia | Under treatment | \ | re-mission video game | Internet | Participants used the Re-Mission video game, designed to improve the physical and psychological health of cancer patients. Each session lasted 10-15 minutes, followed by a one-hour rest period, with total daily gameplay time not exceeding one hour. | 3 months | scale for the assessment of fatigue in pediatric oncology patients aged 13–18 | At the 1- and 3-month assessments, adolescents in the experimental group had significantly higher scores than those in the control group across general fatigue, sleep/rest fatigue, cognitive fatigue, and treatment-related concerns (*P*<.001), indicating a marked improvement in fatigue levels. | Physical function, emotional well-being |
| **Robot-Assisted Intervention** | |  |  |  |  |  |  |  |  |  |  |  |  |  |  |  |
|  | | Atkinson 2023 [29] | Australia | Quasi-experimental study | 5–25 | 21 | Various types (Sarcoma, Leukemia, Lymphoma, Central nervous system germ cell tumor, Medulloblastoma, Pilocytic astrocytoma, Sacral meningioma-papillary variant, Myxopapillary ependymoma) | Undergoing or completed cancer treatment | \ | Lokomat and ArmeoSpring rehabilitation robot | Robotic Systems | Participants engaged in training with the Lokomat or ArmeoSpring rehabilitation robots, targeting gait or upper limb function, respectively, with robot selection based on clinical needs. Neither type of training was conducted simultaneously within the same cycle. Training sessions were held twice a week for 6 weeks, with each session lasting at least 20 minutes, gradually increasing to 40 minutes. | 6 weeks | FACIT-F^f^ | Based on the FACIT-F scale results, the effect size for the Lokomat robot intervention was *r*=0.53, with a *P*-value of .04. For the ArmeoSpring robot intervention, due to the small sample size (n=3), only the pre- and post-intervention effect size was calculated, which was *r*=0.62. | Physical function |
|  | a RAND-36: RAND 36-Item Health Survey  b PedsQL multidimensional fatigue scale: Pediatric quality-of-life inventory multidimensional fatigue scale  c PROPr: patient-reported outcomes measurement information system—Preference  d FSI 15-item: Fatigue Symptom Inventory 15-item  e POMS: Profile of Mood States  f FACIT-F: Functional Assessment of Chronic Illness Therapy-Fatigue Scale  g CIS20: Checklist Individual Strength 20 | | | | | | | | | | | | | | | |
